# Enhanced detection of polymicrobial bloodstream infections with 16S rDNA PCR library amplicons and clinical relevance

**DOI:** 10.3389/fmicb.2026.1916658

**Published:** 2026-10-15

**Authors:** Guangqi Li, Geng Wu, He Yin, Kendall A. McGraw, Jia Hou, Zhijun Zhao

**Affiliations:** 1 Medical Laboratory Center, General Hospital of Ningxia Medical University, Yinchuan, Ningxia, China; 2 Ningxia Key Laboratory of Clinical Pathogenic Microorganisms, Yinchuan, Ningxia, China; 3 Medical Laboratory Center, Xiamen Humanity Women and Children’s Hospital, Xiamen, Fujian, China; 4 Biomedical Science, College of Medicine, The Ohio State University, Columbus, OH, United States; 5 Department of Pulmonary and Critical Care Medicine, General Hospital of Ningxia Medical University, Yinchuan, Ningxia, China; 6 Central Laboratory, Peking University First Hospital Ningxia Women and Children’s Hospital (Ningxia Hui Autonomous Region Maternal and Child Health Hospital), Yinchuan, Ningxia, China

**Keywords:** 16S rDNA PCR library amplicons, blood culture, clinical significance, high-throughput sequencing, polymicrobial bloodstream infection

## Abstract

**Introduction:**

Polymicrobial bloodstream infections (BSIs) are clinically challenging because conventional blood culture may fail to recover all causative microorganisms, particularly in patients with low microbial burden, prior antimicrobial exposure, or infections caused by fastidious organisms. This study evaluated a 16S rDNA PCR library construction strategy based on cloning and Sanger sequencing as a complementary approach to conventional blood culture and compared its microbial detection profile with that of direct 16S rDNA amplicon high-throughput sequencing.

**Methods:**

We retrospectively analyzed 16,970 blood culture specimens collected at a hospital in northwestern China to characterize the clinical distribution and epidemiological features of BSIs. In a retrospective cohort of 295 patients with culture-confirmed BSI, clinical predictors associated with blood culture-defined polymicrobial BSI were evaluated using multivariable logistic regression and receiver operating characteristic (ROC) curve analyses, with particular emphasis on white blood cell (WBC) count and procalcitonin (PCT) levels. In a separate molecular comparison cohort of 20 patients, cell-free DNA (cfDNA) was analyzed using two 16S rDNA-based workflows: a clone-based approach involving PCR amplification, gel purification, cloning, amplified ribosomal DNA restriction analysis (ARDRA), and Sanger sequencing, and a direct 16S rDNA amplicon high-throughput sequencing approach.

**Results:**

Among the 16,970 blood culture specimens, 1,321 were positive, including 45 polymicrobial BSI cases. *Escherichia coli* was the most frequently identified pathogen, followed by coagulase-negative staphylococci and *Brucella* spp. Compared with monomicrobial BSI, polymicrobial BSI was associated with higher WBC counts and PCT levels. In multivariable analysis, both WBC count and PCT were independently associated with polymicrobial BSI, and their combination showed good discriminatory performance (AUC = 0.927). In the separate molecular comparison cohort of 20 patients, the three methods showed distinct detection ranges. Blood culture detected 0–1 microbial taxon per patient, with one pathogen identified in 17 patients and negative results in 3 patients. The clone-based method detected a broader microbial spectrum, identifying 0–5 taxa per patient, whereas direct 16S rDNA amplicon sequencing showed the broadest detection range, with more than five taxa detected in 9 patients. In contrast, 16S rDNA high-throughput sequencing generated broader taxonomic profiles but showed lower agreement with blood culture. Compared with conventional blood culture, the 16S rDNA PCR library method showed an overall percent agreement of 80.0%.

**Conclusion:**

WBC and PCT may help identify patients at increased risk of polymicrobial BSI and may support the selection of patients for additional molecular testing. The cloning-based 16S rDNA PCR library strategy provides a complementary approach to conventional blood culture and may offer a balance between broader microbial detection and more focused sequence-level interpretation compared with direct amplicon high-throughput sequencing. Its potential utility may be particularly relevant in selected patients with culture-negative or clinically suspected polymicrobial BSI.

## Introduction

BSIs are life-threatening conditions characterized by systemic inflammatory responses triggered by pathogenic microorganisms in the blood (Evans et al., 2021). These infections often lead to extensive inflammatory cascades, frequently progressing to sepsis and multiple organ dysfunction syndrome (MODS), posing a substantial global public health burden (Cheng et al., 2025). Polymicrobial BSIs involve more than one pathogen and are associated with higher mortality rates, increased treatment failure, and greater clinical complexity than monomicrobial infections (Lee et al., 2022).

Timely and comprehensive identification of the infectious source and pathogen spectrum is critical for optimizing antimicrobial therapy. Blood culture (BC) is a routine reference method for the diagnosis of bloodstream infections, providing viable organisms for identification and antimicrobial susceptibility testing. Modern automated BC systems have significantly reduced the time to detection, often showing growth within 24 h (Gatti et al., 2023). Furthermore, matrix-assisted laser desorption/ionization time-of-flight mass spectrometry (MALDI-TOF MS) has revolutionized microbial identification due to its speed and cost-effectiveness once colonies are isolated (Calderaro and Chezzi, 2024). BC results may be biased toward dominant or fast-growing bacteria, potentially masking low-abundance, fastidious, or slow-growing organisms, especially in patients who have received prior antimicrobial treatment. Additionally, slow-growing bacteria may take 3–7 days to become detectable, delaying the timely initiation of aggressive treatment (Fenollar and Raoult, 2007). While MALDI-TOF MS is efficient for single isolates, it cannot directly characterize mixed bacterial populations from a single positive culture without prior subculturing, potentially delaying recognition of complex infection patterns.

Molecular diagnostics, such as 16S rDNA high-throughput sequencing, offer broad-range detection and high sensitivity. However, their routine clinical application is often hampered by high costs, the need for complex bioinformatics support, and the difficulty of distinguishing true pathogens from environmental contaminants and background signals (Xu et al., 2025). As a robust alternative, 16S rDNA PCR library amplicons are generated by amplifying bacterial 16S rDNA with universal primers, followed by molecular cloning and sequencing. By segregating individual rDNA sequences into clones, this approach enables the dissection of mixed microbial signals that would otherwise yield uninterpretable results with traditional Sanger sequencing (Chen et al., 2022, Schabereiter-Gurtner et al., 2001). 16S rDNA PCR library amplicons have demonstrated high reproducibility and sensitivity in microbial ecology, providing a framework to explore bacterial diversity beyond conventional culture-based methods.

In this study, we aim to evaluate the role of 16S rDNA PCR library amplicons as a complementary diagnostic approach for polymicrobial BSI. By integrating retrospective clinical data, inflammatory biomarkers, and parallel pathogen detection using BC, 16S rDNA PCR library amplicons, and 16S rDNA high-throughput sequencing, we aim to determine whether this strategy could improve characterization of microbial complexity in BSIs and provide clinically useful adjunctive microbiological information.

## Materials and methods

### Study design and patient recruitment

This study was conducted at a hospital in Northwest China, and comprised three complementary components. First, a 1-year retrospective epidemiological analysis was performed on 16,970 BC specimens processed from January to December 2024 to characterize the local epidemiological patterns and pathogen distribution of BSIs. Second, to obtain a sufficient number of polymicrobial BSI cases given their relatively low detection frequency, the study period for the retrospective clinical cohort was extended from January 2022 to December 2024. During this period, 300 culture-positive BSI cases were initially identified, including all eligible polymicrobial BSI cases and randomly selected monomicrobial BSI cases. After excluding 3 cases meeting Clinical and Laboratory Standards Institute (CLSI) contamination criteria and 2 with incomplete clinical data, 295 patients were included in the analysis of clinical and inflammatory markers associated with polymicrobial BSI. Third, a prospective molecular comparison was conducted in patients with suspected BSI during the second quarter of 2024. Two cases were randomly selected each week, yielding 24 initially selected cases. Four cases were subsequently excluded because of incomplete clinical information or failure of cfDNA extraction, resulting in a final molecular comparison cohort of 20 patients. These patients underwent parallel conventional BC, 16S rDNA PCR library construction, and 16S rDNA high-throughput sequencing, and their detailed clinical and microbiological characteristics are presented in Supplementary Table 1.

### Inclusion and exclusion criteria

Patients were initially screened according to predefined clinical criteria for suspected BSI, including a body temperature of ≥ 38°C or < 36°C accompanied by systemic toxic symptoms, hypotension, or laboratory evidence of infection. Patients with laboratory-confirmed BSI who met the study eligibility criteria were included in the retrospective clinical predictor cohort. To minimize confounding of inflammatory-marker analyses, patients with autoimmune diseases, hematologic disorders, or ongoing radiotherapy, chemotherapy, or long-term immunosuppressive therapy were excluded. These exclusion criteria were applied specifically to the inflammatory-marker analysis and did not constitute general exclusion criteria for molecular testing.

Only the first eligible BSI episode per patient was included in the retrospective analysis, and repeated isolation of the same microorganism during the same infectious episode was considered a single microbiological event. Polymicrobial BSI was defined as the recovery of two or more clinically relevant bacterial species from blood cultures obtained during the same infectious episode, after exclusion of organisms considered contaminants according to the predefined laboratory criteria.

### Specimen collection and laboratory processing

For microbiological diagnosis, 8–10 mL of peripheral venous blood was collected aseptically and inoculated into paired aerobic and anaerobic culture bottles. The bottles were incubated using the BacT/ALERT VIRTUO system (bioMérieux, France). Upon positive alert, samples were subcultured onto blood agar plates and incubated at 37°C for 18–36 h. Species identification of isolated colonies was subsequently performed using Vitek MALDI-TOF MS.

Concurrent with standard BC collection, an additional 3–5 mL of peripheral venous blood was obtained for molecular analysis. Plasma was separated by centrifugation at 3,500 × g for 20 min. Genomic DNA was extracted using a bacterial DNA extraction kit (GensKey Medical Technology, Tianjin, China). To monitor contamination, a sterile, DNA-free water sample served as a “no-template” negative control and was processed in parallel with clinical samples throughout all extraction and amplification steps. For assay validation, reference strains representing the three most frequently isolated pathogens identified by our statistical analysis—specifically *Escherichia coli*, *Staphylococcus aureus*, and coagulase-negative staphylococci—were cultured overnight in LB broth at 37°C with agitation. Extracted genomic DNA from these strains was spiked into DNA solutions derived from negative control plasma to create standardized positive controls representing both monomicrobial and distinct polymicrobial infection profiles.

### 16 S rDNA PCR amplification, library construction, and sequence analysis

cfDNA was extracted from 600 μL of plasma using a nucleic acid extraction kit (GensKey Medical Technology, Tianjin, China). Broad-range bacterial 16S rDNA was amplified using universal primers 16F (5’-AGAGTTTGATCCTGGCTCAG-3’) and 1492R (5’-GGTTACCTTGTTACGACTT-3’). Following purification using a gel extraction kit (TIANGEN, Beijing, China), the amplicons were ligated into T5 cloning vectors and transformed into *Escherichia coli* DH5α competent cells. Positive clones were screened via colony PCR with M13 primers and subsequently subjected to amplified ribosomal DNA restriction analysis (ARDRA) using the restriction enzymes HhaI and RsaI (Takara, Japan). Clones displaying identical restriction fragment patterns were clustered into operational taxonomic units (OTUs). Representative clones from each OTU were sequenced (Tsingke Biotechnology, Beijing, China), and taxonomic classification was determined by BLAST alignment against the NCBI GenBank database.

### 16 S rDNA high-throughput sequencing

cfDNA was extracted from plasma samples and quantified using a Qubit fluorometer. For amplicon library preparation, 30 ng of cfDNA was subjected to PCR amplification using fusion primers containing the target-specific 16S rDNA sequences and sequencing adapter/index sequences. The resulting PCR products were purified using Agencourt AMPure XP magnetic beads and evaluated for fragment size and concentration using an Agilent 2100 Bioanalyzer. Qualified libraries were subsequently subjected to high-throughput sequencing on an Illumina HiSeq platform (BGI Genomics, China). In contrast to the clone-based approach, this method directly subjected PCR-amplified 16S rDNA products to high-throughput sequencing, without an intermediate cloning and colony-screening step. Sequencing data were processed using a standard bioinformatics workflow, including quality control and filtering of raw FASTQ reads, sequence assembly, OTU clustering, and taxonomic annotation. During data analysis, known laboratory and skin-associated contaminants were excluded, and the positive detection criteria recommended by BGI (China) based on its internal analytical performance evaluation and clinical testing validation were adopted, i.e., a relative abundance threshold > 0.05.

### Complete blood count and procalcitonin testing

Venous blood samples (1–3 mL) were collected into K2-EDTA anticoagulant tubes and analyzed within 2 h. CBC was performed using the XN-9000 automated hematology analyzer (Sysmex, Japan). Procalcitonin (PCT) concentrations were measured using the AQT90 FLEX analyzer (Radiometer, Denmark). Hematological parameters, including white blood cell count (WBC), neutrophil percentage (NEUT%), absolute neutrophil count (ANC), lymphocyte count (LYM), monocyte count, platelet count (PLT), and red cell distribution width (RDW) were recorded. Derived indices, including neutrophil-to-lymphocyte ratio (NLR), lymphocyte-to-monocyte ratio (LMR), platelet-to-lymphocyte ratio (PLR), neutrophil-to-platelet ratio (NPR), systemic immune-inflammation index (SII), and systemic inflammatory response index (SIRI), were calculated accordingly.

### Clinical and statistical analysis

Positive BC cases were analyzed for pathogen distribution and polymicrobial infection rates. Clinical and laboratory parameters were compared between monomicrobial and polymicrobial infection groups, as well as among Gram-positive, Gram-negative, and mixed Gram stain subgroups. Multivariate logistic regression was used to identify independent factors associated with polymicrobial BSI. Receiver operating characteristic (ROC) curve analysis was used to evaluate the diagnostic performance of individual and combined biomarkers.

Statistical analyses were conducted using SPSS version 25.0. Continuous variables were reported as mean ± SD or median, as appropriate, and compared using Student’s t test or the Mann–Whitney U test. Categorical variables were compared using the chi-square test. Diagnostic performance metrics for the molecular methods, including concordance and sensitivity, were assessed against BC as the reference standard. A two-sided *P* < 0.05 was considered statistically significant.

## Results

### Pathogen distribution and epidemiological characteristics

During the 1-year surveillance period (January to December 2024), a total of 16,970 BC specimens were processed. Of these, 1,321 (7.8%) were positive for microbial growth. Based on standard culture criteria, most positive cases were classified as monomicrobial infections (96.6%), while polymicrobial BSIs accounted for 3.4%. As illustrated in Figure 1A, the pathogen spectrum was dominated by *Escherichia coli*, followed by coagulase-negative staphylococci and *Brucella* spp.

**FIGURE 1:**
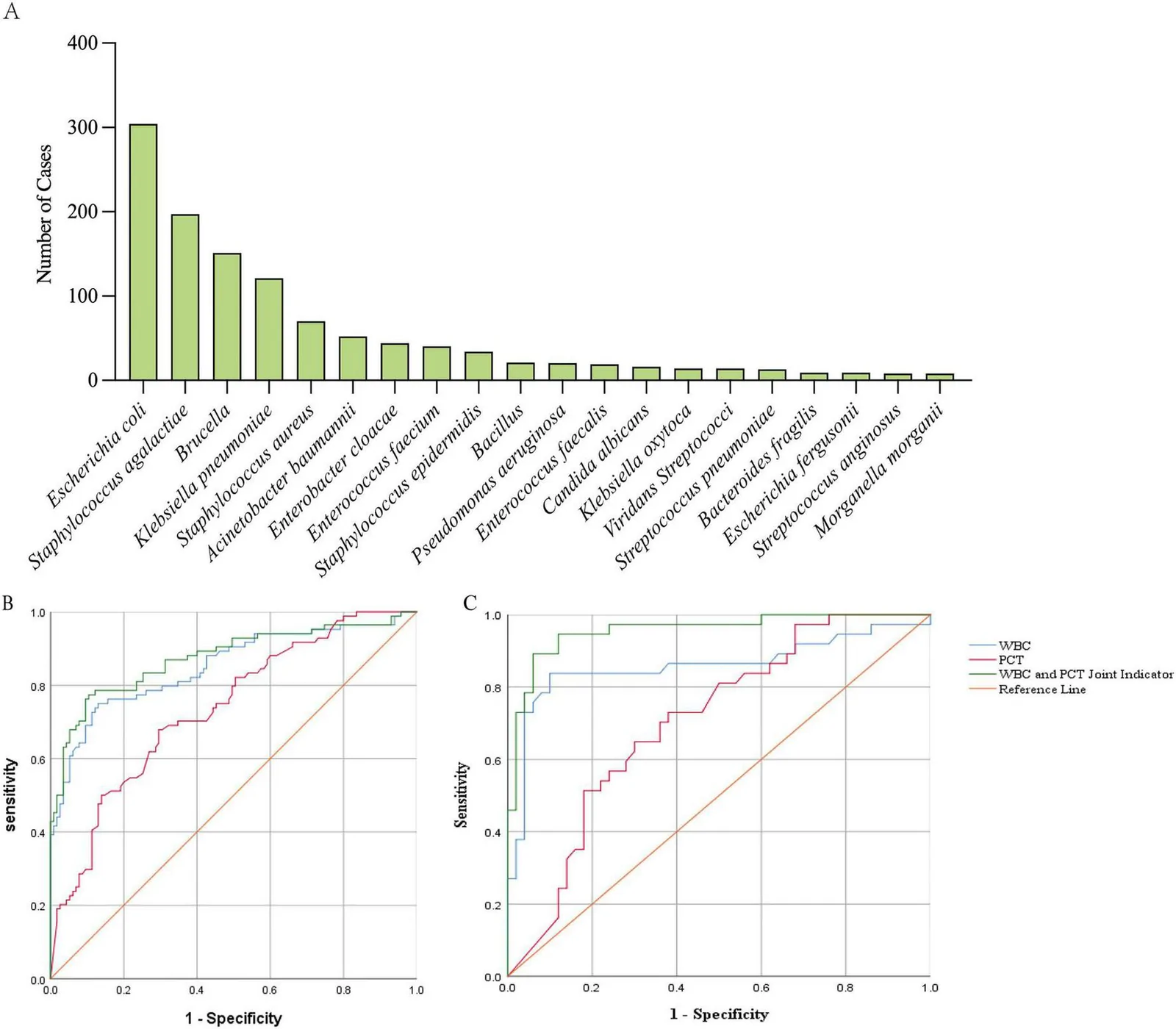
Pathogen distribution and epidemiological characteristics. **(A)** Distribution of the top 20 bacterial pathogens identified within the annual blood culture results. **(B)** ROC curve of clinical indicators for predicting polymicrobial bloodstream infection. **(C)** ROC curve of clinical indicators for predicting mixed Gram-type polymicrobial bloodstream infections.

### Features of polymicrobial infection

Next, to evaluate clinical indicators associated with polymicrobial infection, we constructed a retrospective cohort of 300 culture-positive BSI cases including all polymicrobial cases identified over 3 years (*n* = 180) and randomly selected monomicrobial cases. After excluding three cases per CLSI contamination criteria and two additional cases with incomplete clinical data, 295 patients were included in the final analysis. Demographic characteristics did not differ significantly between groups (age *P* = 0.322; sex *P* = 0.142).

A total of 491 bacterial isolates were identified in the 295-patient cohort, including 292 Gram-negative (59.5%) and 199 Gram-positive (40.5%). The most prevalent Gram-negative organisms were *Escherichia coli* (25.7%) and *Klebsiella pneumoniae* (38/292, 13.0%), whereas the most common Gram-positive organisms were *Enterococcus faecalis* (51/199, 25.6%) and coagulase-negative staphylococci (24/199, 12.1%). Among polymicrobial infections, 17.3% involved only Gram-positive organisms, 32.7% involved only Gram-negative, and 50.0% involved mixed Gram types.

### Association of polymicrobial BSI with inflammatory markers

Compared with monomicrobial BSI, polymicrobial BSI was associated with significantly higher systemic inflammatory markers, including WBC, NEUT%, ANC, MXD, NLR, NPR, SII, SIRI, and PCT (all *P* < 0.05; Tables 1, 2). Among polymicrobial infections, mixed Gram-type infections showed higher levels of these markers than single Gram-type infections (*P* < 0.05), suggesting a greater inflammatory burden in more complex infection patterns.

**TABLE 1:** Comparison of clinical indicators between monomicrobial and polymicrobial bloodstream infection.

Groups	n	WBC(*10^9^/L)	NEUT%	ANC(*10^9^/L)	MXD(*10^9^/L)	NPR
Monobacterial infection	115	10.13(8.37, 11.32)	83.60(76.50, 88.80)	8.09(6.89, 9.60)	0.60(0.37, 0.86)	0.04(0.03, 0.09)
Polymicrobial infection	180	15.18(12.66, 21.36)	89.30(83.78, 93.18)	13.48(10.45, 19.74)	0.69(0.47, 1.00)	0.11(0.05, 0.24)
Statistic		−11.230	−5.57	−11.242	−2.327	−6.96
*P-*value		< 0.001	<0.001	< 0.001	0.020	< 0.001
**Groups**	**n**	**NLR**	**SII**	**SIRI**	**PCT(ng/mL)**	
Monobacterial infection	115	9.53(5.58, 15.20)	1492.56(765.19, 2405.46)	4.53(3.09, 9.22)	1.40(0.63, 3.00)	
Polymicrobial infection	180	15.58(9.62, 29.24)	2227.96(1201.48, 4301.75)	10.18(5.66, 19.00)	14.00(6.65, 34.00)	
Statistic		−5.661	−3.531	−6.559	−10.791	
*P-*value		< 0.001	<0.001	< 0.001	<0.001	

Data that conform to normal distribution are expressed as Mean ± SD with the statistic t. Indicators that do not conform to normal distribution are expressed as M (P25, P75) with the statistic Z.

**TABLE 2:** Comparison of clinical indicators between single Gram-type and mixed Gram-type polymicrobial bloodstream infection.

Groups	n	WBC(*10^9^/L)	NEUT%	ANC(*10^9^/L)	NPR	NLR
Single Gram-type polymicrobial BSI	90	12.79(11.06, 14.05)	86.80(80.35, 91.13)	10.52(9.22, 12.82)	0.07(0.04, 0.13)	12.00(8.21, 20.70)
Mixed Gram-type polymicrobial BSI	90	20.75(17.45, 28.51)	91.40(86.60, 93.83)	18.96(15.10, 25.52)	0.16(0.08, 0.38)	19.79(12.89, 38.94)
Statistic		−9.936	−4.667	−9.600	−4.921	−4.157
*P-*value		< 0.001	<0.001	< 0.001	<0.001	< 0.001
**Groups**	**n**	**SII**		**SIRI**		**PCT(ng/mL)**
Single Gram-type polymicrobial BSI	90	1909.69(1002.95, 3657.52)		8.04(4.60, 11.84)		7.35(3.23, 12.25)
Mixed Gram-type polymicrobial BSI	90	2612.27(1337.46, 5121.03)		13.93(6.83, 31.67)		31.00(15.50, 47.50)
Statistic		−2.214		−4.537		−8.327
*P-*value		0.027		< 0.001		<0.001

Data that conform to normal distribution are expressed as Mean ± SD with the statistics. Indicators that do not conform to normal distribution are expressed as M (P25, P75) with the statistic Z.

### WBC and PCT are independent predictors for polymicrobial BSI

Variables significant in univariate analysis—including WBC, NEUT%, MXD, NLR, NPR, SII, SIRI, and PCT—were entered into logistic regression models. Elevated WBC (OR = 1.723, 95%CI 1.472–2.017, *P* < 0.001) and PCT (OR = 1.028, 95%CI 1.003–1.054, *P* = 0.031) independently predicted polymicrobial BSI (Table 3). For Gram-type BSI, WBC (OR = 1.486, 95%CI 1.292–1.710, *P* < 0.001) and PCT (OR = 1.042, 95%CI 1.017–1.068, *P* = 0.001) were independently associated with mixed Gram-type infections (Table 4).

**TABLE 3:** Binary logistic regression analysis of independent risk factors for developing mixed bacterial bloodstream infections.

Laboratory marker	*B*	SE	*P-*value	OR(95% CI)
WBC	0.544	0.080	< 0.001	1.723(1.472∼2.017)
NEUT%	−0.022	0.044	0.618	0.978(0.897∼1.067)
ANC	0.183	0.388	0.636	1.201(0.562∼2.569)
NLR	−0.006	0.022	0.797	0.994(0.952∼1.038)
NPR	0.807	0.681	0.236	2.241(0.591∼8.506)
SII	0.000	0.000	0.736	1.000(1.000∼1.000)
SIRI	−0.013	0.036	0.717	1.013(0.944∼1.087)
MXD	−0.470	0.322	0.145	0.625(0.332∼1.176)
PCT	0.027	0.013	0.031	1.028(1.003∼1.054)

**TABLE 4:** Binary logistic regression analysis of independent risk factors for developing mixed gram stained bacterial bloodstream infections.

Laboratory marker	*B*	SE	*P-*value	OR(95%CI)
SIRI	−0.045	0.034	0.194	0.956(0.894∼1.023)
WBC	0.396	0.072	< 0.001	1.486(1.292∼1.710)
NEUT%	−0.017	0.046	0.702	0.983(0.899∼1.075)
ANC	0.032	0.109	0.768	1.033(0.834∼1.279)
NLR	0.024	0.022	0.280	1.024(0.981∼1.070)
SII	0.000	0.000	0.393	1.000(1.000∼1.000)
NPR	0.075	0.465	0.871	1.078(0.433∼2.683)
PCT	0.041	0.013	0.001	1.042(1.017∼1.068)

Univariate analysis indicated that both WBC counts and PCT levels were significantly higher in the polymicrobial group compared to the monomicrobial group. Using monomicrobial infections as controls, ROC analysis demonstrated that WBC alone achieved an AUC of 0.890 (95%CI 0.853∼0.928) with 77.1% sensitivity and 90.0% specificity at 12.54 × 10^9^/L, while PCT alone achieved an AUC of 0.910 (95%CI 0.875∼0.945) with 88.0% sensitivity and 80.0% specificity at 3.20 ng/mL (Figures 1B,C and Tables 5, 6). Combining WBC and PCT improved discriminative performance (AUC 0.927, 95%CI 0.897∼0.957), with balanced sensitivity (84.0%) and specificity (91.8%), supporting their potential utility in identifying polymicrobial and mixed Gram-type BSI.

**TABLE 5:** The value of clinical indicators in identifying polymicrobial bacterial bloodstream infections.

Risk factors	AUC(95%CI)	Cut-off value	SE	*P*	Sensitivity	Specificity	Jordon index
WBC	0.890(0.853∼0.928)	12.54*10^9^/L	0.019	< 0.001	77.1%	90.0%	0.671
PCT	0.910(0.875∼0.945)	3.20 ng/mL	0.018	< 0.001	88.0%	80.0%	0.680
Combined indicators	0.927(0.897∼0.957)	\	0.015	< 0.001	84.0%	91.8%	0.758

**TABLE 6:** The value of clinical indicators in identifying mixed Gram staining results bacterial bloodstream infections.

Risk factors	AUC(95%CI)	Cut-off value	SE	*P*	Sensitivity	Specificity	Jordon index
WBC	0.932(0.894∼0.970)	16.31*10^9^/L	0.019	< 0.001	85.1%	93.2%	0.783
PCT	0.868(0.814∼0.921)	13.50 ng/mL	0.027	< 0.001	81.6%	78.4%	0.600
Combined indicators	0.950(0.917∼0.983)	\	0.017	< 0.001	94.30%	89.80%	0.841

### FIG1 pathogen distribution and epidemiological characteristics

A: Distribution of the top 20 bacterial pathogens identified within the annual blood culture results. B: ROC curve of clinical indicators for predicting polymicrobial bloodstream infection. C: ROC curve of clinical indicators for predicting mixed Gram-type polymicrobial bloodstream infections.

### Comparison of the 16S rDNA PCR library amplicons versus blood culture for detecting polymicrobial infection

Broad-range amplification of bacterial 16S rDNA using universal primers produced amplicons of approximately 1,500 bp, consistent with the expected near-full-length 16S rRNA gene. Agarose gel electrophoresis confirmed specific amplification with no detectable bands in negative controls (Figure 2A). Purified amplicons were successfully ligated into cloning vectors and transformed into competent *Escherichia coli* cells. ARDRA using HhaI and RsaI generated distinct restriction fragment patterns (Figure 2B), allowing cloned inserts to be grouped into OTUs. Representative clones from each OTU were selected for Sanger sequencing. Sequence alignment against the NCBI GenBank database demonstrated ≥ 95% similarity to reference sequences, enabling species-level taxonomic assignment.

**FIGURE 2:**
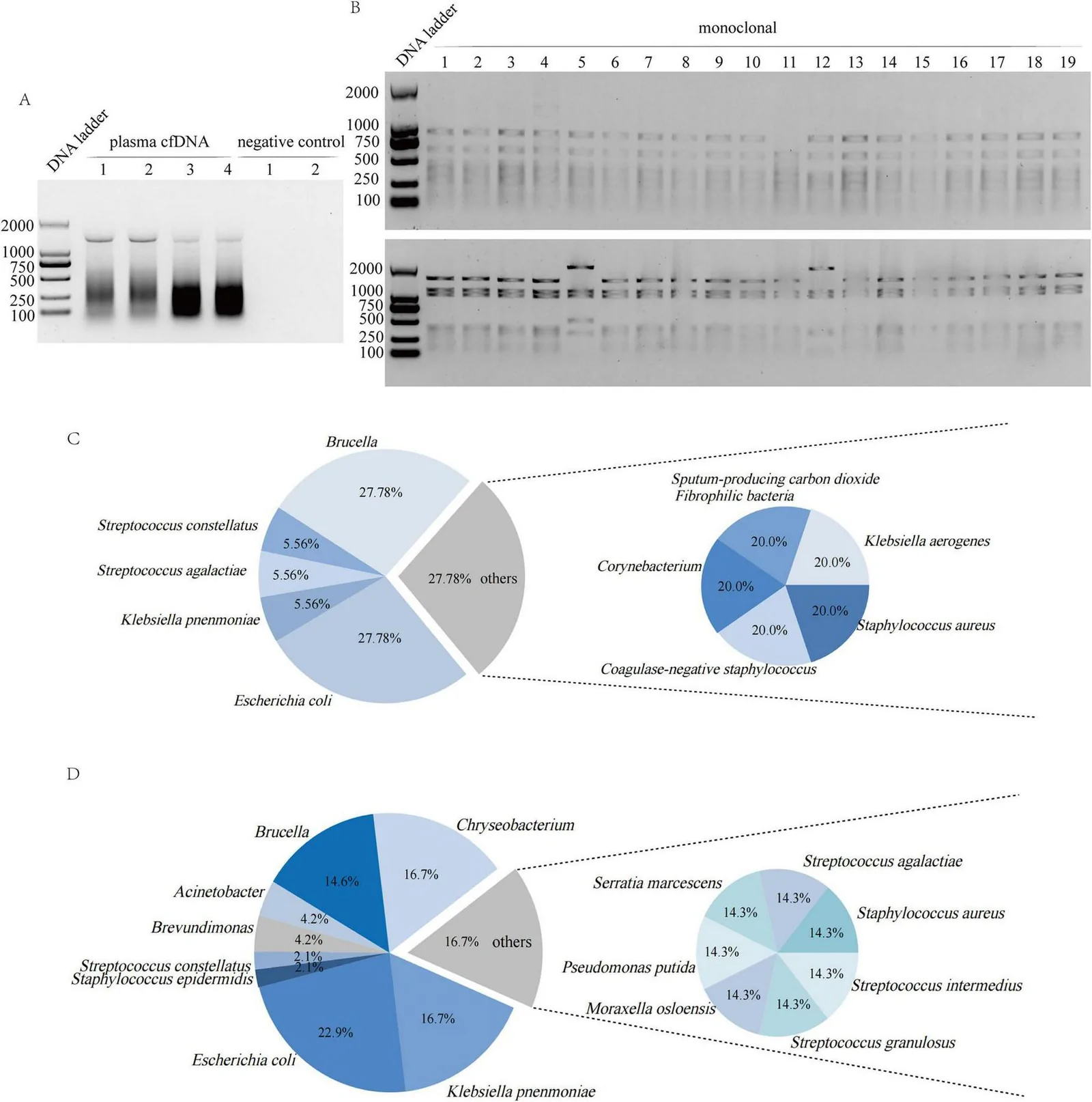
The bacterial 16S rDNA amplification results. **(A)** Bacterial 16S rDNA PCR amplification results. **(B)** Bacterial 16S rDNA restriction enzyme digestion results (RsaI digestion on top, HhaI digestion on bottom). **(C)** Species distribution detected by blood culture technology. **(D)** Species distribution detected by 16S rDNA library construction technology.

In the subset of 20 patients analyzed by molecular methods, blood culture identified 10 bacterial species, with *Escherichia coli* and *Brucella* being the most frequently detected organisms (Figure 2C). In contrast, 16S rDNA library construction identified a total of 16 bacterial species, including *Escherichia coli*, *Klebsiella pneumoniae*, and *Chryseobacterium chrysogenum* as the most detected taxa (Figure 2D and Supplementary Table 2).

Blood culture detected 0–1 microbial taxon per patient, with one pathogen identified in 17 patients and negative results in 3 patients. The clone-based method detected a broader microbial spectrum, identifying 0–5 taxa per patient. Concordance analysis between the two methods classified results as completely consistent in 6 cases (30%), partially consistent in 10 cases (50%), and inconsistent in 4 cases (20%). Mixed bacterial signals, defined as the detection of two bacterial species in a single sample by the 16S method, were observed in all 20 samples. Overall, 16S rDNA PCR library construction preserved agreement with the dominant pathogens identified by BC while revealing additional co-detected organisms in several cases.

### FIG2 The bacterial 16S rDNA amplification results

A: Bacterial 16S rDNA PCR amplification results. B: Bacterial 16S rDNA restriction enzyme digestion results (RsaI digestion on top, HhaI digestion on bottom). C: Species distribution detected by blood culture technology. D: Species distribution detected by 16S rDNA library construction technology.

### High-throughput sequencing in detecting polymicrobial infections

Among the 20 analyzed patients, 16S rDNA high-throughput sequencing detected multiple taxa with relative abundance > 0.05, indicating broader bacterial profiles than those obtained by BC alone. At the genus level, the most abundant taxa included *Escherichia* (23.3%), *Staphylococcus* (14.0%), and *Streptococcus* (18.6%).

Compared with 16S rDNA PCR library amplicons, high-throughput sequencing detected a greater number of bacterial taxa per patient, with identification of additional taxa such as *Streptococcus*, *Staphylococcus*, *Brevundimonas*, and *Acinetobacter* (Figure 3 and Supplementary Table 3). Method comparison revealed no instances of complete concordance, however, 90% showed hierarchical inclusion, in which high-throughput sequencing detected all species identified by the conventional 16S method and identified additional taxa not captured by culture or by 16S rDNA PCR library amplicons. Due to the lack of standardized diagnostic thresholds for high-throughput sequencing, interpreting these additional findings in a clinical context remains challenging. Taken together, these data reveal comprehensive pathogen diversity and methodological discordance of high-throughput sequencing in detecting polymicrobial infections.

**FIGURE 3:**
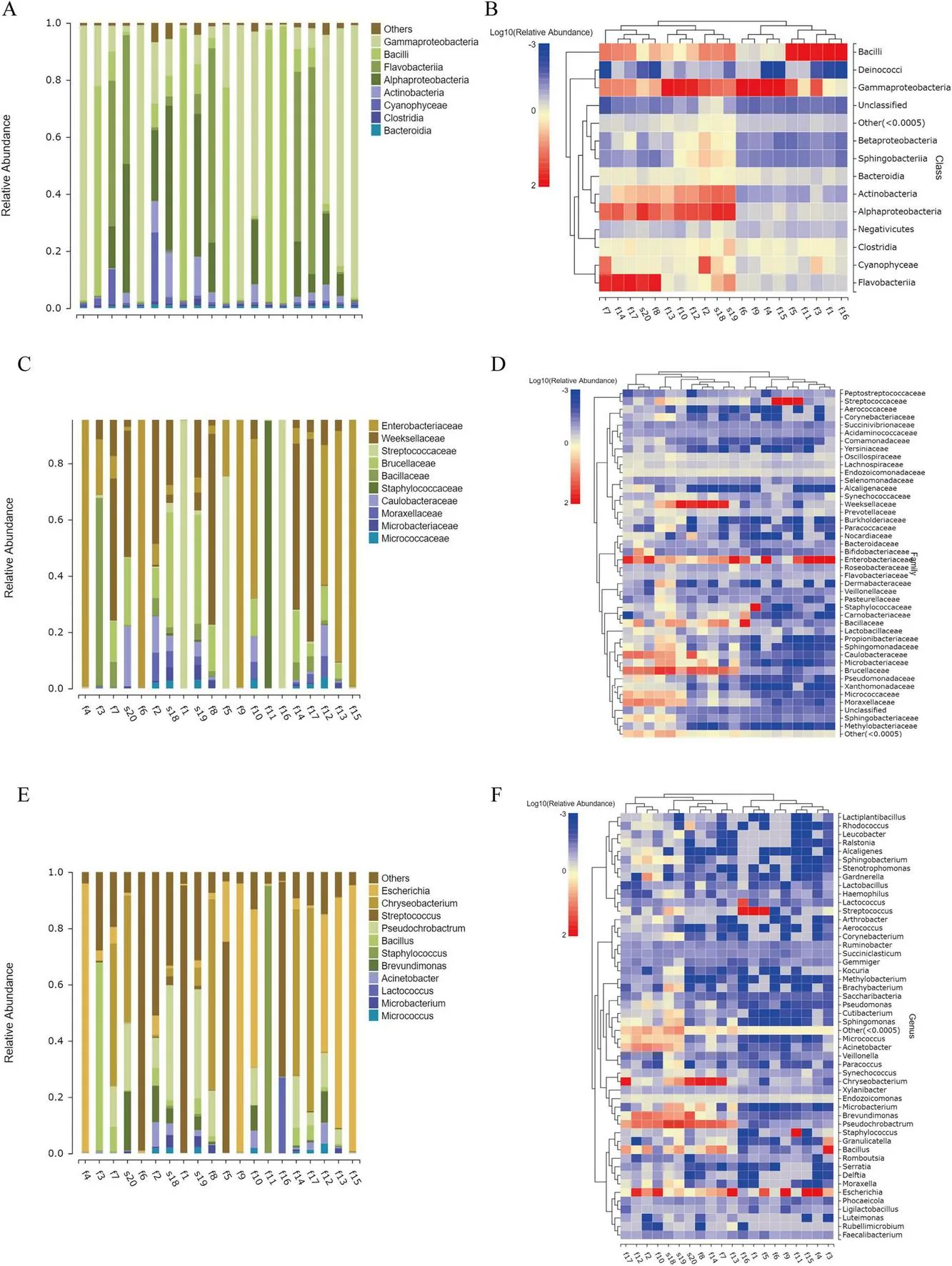
High-throughput sequencing results of 16S rDNA for each sample. **(A)** Species distribution detected by 16S rDNA high-throughput sequencing (classified to the phylum level). **(B)** Heatmap of species distribution detected by 16S rDNA high-throughput sequencing (phylum level). **(C)** Species distribution detected by 16S rDNA high-throughput sequencing (family level). **(D)** Heatmap of species distribution detected by 16S rDNA high-throughput sequencing family level). **(E)** Species distribution detected by 16S rDNA high-throughput sequencing (genus level). **(F)** Heatmap of species distribution detected by 16S rDNA high-throughput sequencing (genus level).

### FIG3 high-throughput sequencing results of 16S rDNA for each sample

A: Species distribution detected by 16S rDNA high-throughput sequencing (classified to the phylum level). B: Heatmap of species distribution detected by 16S rDNA high-throughput sequencing (phylum level). C: Species distribution detected by 16S rDNA high-throughput sequencing (family level). D: Heatmap of species distribution detected by 16S rDNA high-throughput sequencing family level). E: Species distribution detected by 16S rDNA high-throughput sequencing (genus level). F: Heatmap of species distribution detected by 16S rDNA high-throughput sequencing (genus level).

### Consistency of blood culture, 16S rDNA PCR library amplicons, and high-throughput sequencing in detecting polymicrobial infection

Using BC as the reference standard, the consistency and diagnostic performance of 16S rDNA PCR library amplicons and high-throughput sequencing were evaluated. The 16S rDNA PCR library method demonstrated an overall concordance rate of 80.0%, with a sensitivity of 88.2%. High-throughput sequencing showed a concordance rate of 70.0% and a sensitivity of 82.4% (Figure 4).

**FIGURE 4:**
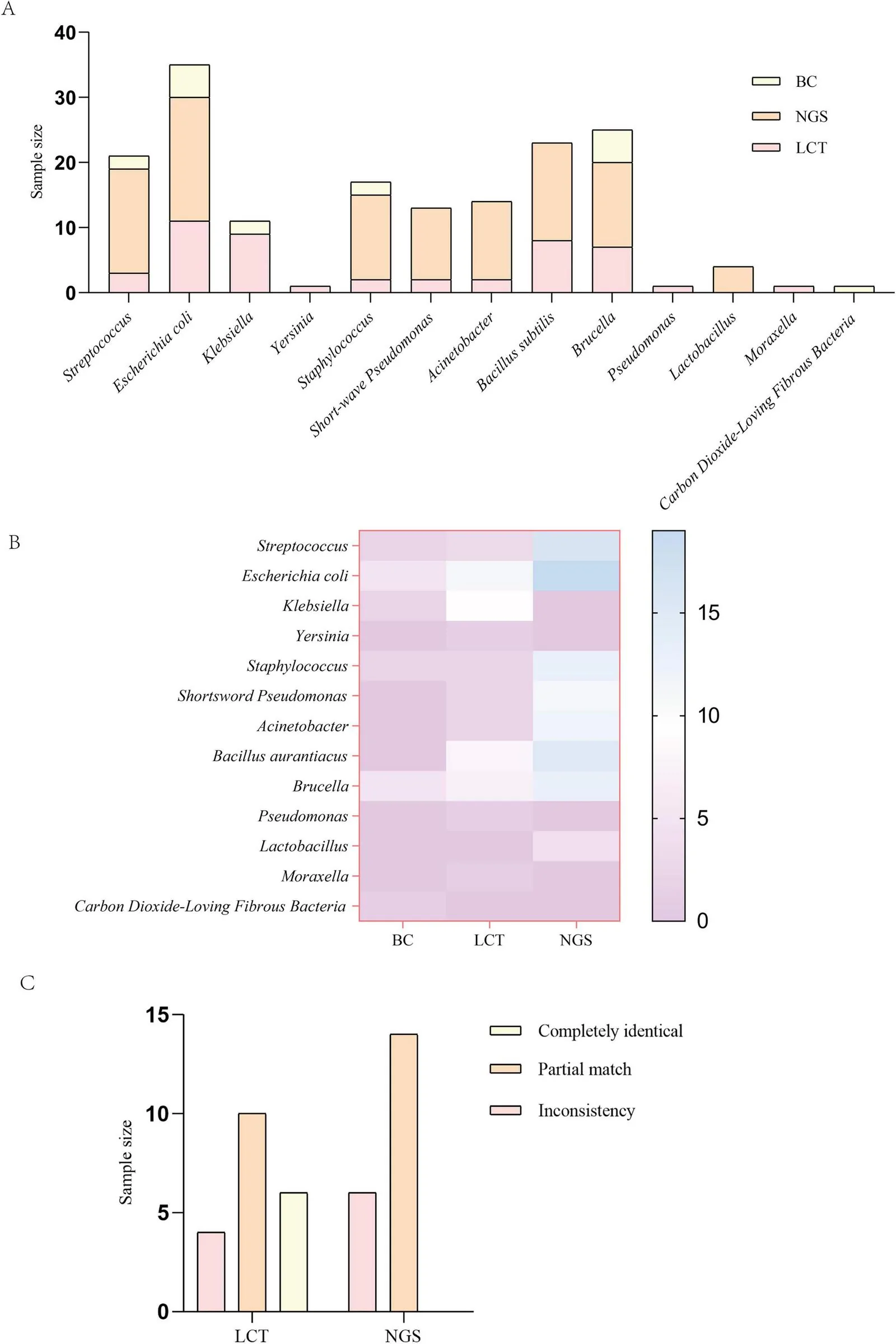
Consistency and diagnostic performance of molecular methods. **(A)** Comparison of pathogen detection results obtained by different molecular diagnostic techniques. **(B)** Heatmap of intergroup comparison of pathogen detection results by different methods. **(C)** Consistency evaluation of detection results of molecular diagnostic techniques. LCT: 16S rDNA library construction technology. NGS: 16S rDNA high-throughput sequencing technology.

To further characterize the differences in microbial detection breadth, the total number of microbial taxa identified by each method was compared across the 20 patients. BC detected no more than one taxon per patient, with three patients showing no detectable organism and 17 patients showing one taxon. In comparison, the 16S rDNA PCR library method identified 0–5 taxa per patient, whereas high-throughput sequencing detected 2 to > 5 taxa per patient, with nine patients yielding more than five taxa (Figure 4). Thus, the PCR library method detected a broader range of microbial taxa than BC but generally fewer taxa than high-throughput sequencing.

The higher concordance of the PCR library method with BC should therefore be interpreted cautiously. The difference may partly reflect the narrower taxonomic yield of the cloning-based approach compared with high-throughput sequencing, which could result in fewer discordant detections relative to BC. Nevertheless, the PCR library method detected additional microbial taxa beyond BC in a substantial proportion of cases, indicating that its higher concordance was not simply attributable to reproducing the limited detection profile of BC. These findings suggest that the cloning-based approach may provide an intermediate detection profile between conventional culture and direct high-throughput sequencing. Further evaluation in larger cohorts with independent clinical reference standards is required to determine whether this concordance translates into improved diagnostic performance or clinical utility.

### FIG4 consistency and diagnostic performance of molecular methods

A: Comparison of pathogen detection results obtained by different molecular diagnostic techniques. B: Heatmap of intergroup comparison of pathogen detection results by different methods. C: Consistency evaluation of detection results of molecular diagnostic techniques. LCT: 16S rDNA library construction technology. NGS: 16S rDNA high-throughput sequencing technology.

## Discussion

BSI poses a critical clinical challenge with substantial morbidity and mortality, particularly among immunocompromised patients, where rapid progression to bacteremia or sepsis necessitates prompt intervention (Sun et al., 2025). Current clinical practice recommends routinely obtaining two sets of blood cultures, consisting of aerobic and anaerobic bottles, without significantly delaying the initiation of antimicrobial therapy (Evans et al., 2021). Blood culture remains a principal microbiological diagnostic method for suspected BSI and provides clinically important information for pathogen identification and antimicrobial susceptibility testing. However, its diagnostic yield can be affected by several factors, including low bacterial burden, prior antimicrobial exposure, and the growth characteristics of individual organisms (Burillo and Bouza, 2014). Although repeat blood cultures are commonly performed when clinically indicated, repeated cultures yield newly identified pathogens in only approximately 2% of cases and may increase the opportunity for contamination (Aiesh et al., 2023), which can complicate clinical interpretation and potentially affect diagnostic decision-making (Tabriz et al., 2004). This diagnostic inadequacy is particularly detrimental in polymicrobial BSI, where conventional methods are prone to overlooking Gram co-infections. In such cases, interpretation of multiple organisms recovered from blood cultures requires integration of microbiological findings with clinical context and laboratory expertise (Doern et al., 2019, Skoglund et al., 2019). Critically, these techniques exhibit poor sensitivity for fastidious organisms such as *Eubacterium lentum* (Fernández Vecilla et al., 2024), as well as biofilm-forming species (Treviño-Rangel et al., 2018), may be more difficult to recover using routine culture approaches, particularly when bacterial loads are low or antimicrobial therapy has been initiated. These characteristics highlight the potential value of complementary molecular approaches for selected patients in whom conventional blood culture results may be negative or incomplete.

The present study provides compelling evidence for the clinical utility of 16S rDNA PCR library amplicons in detecting polymicrobial BSI, a diagnostic challenge frequently encountered in critical care settings. This aligns with prior studies demonstrating the limitations of blood cultures in detecting fastidious or low-abundance pathogens, particularly in immunocompromised patients, where microbial burdens are often suboptimal for culture-based methods (Zhang et al., 2024, Grumaz et al., 2019). The superior sensitivity of 16S rDNA sequencing observed here confirms the findings of Alfonso et al., who reported that 16S rDNA pyrooxygen sequencing detected transient bacteremia after tooth extraction with significantly higher sensitivity than the culture method (Benítez-Páez et al., 2013). In addition, the construction of the 16S rDNA PCR library can serve as an auxiliary diagnostic tool, especially for specific patient groups, such as those with compromised immune function, patients with sepsis who are clinically suspected but have negative blood cultures, patients receiving empirical antibacterial treatment, and cases involving difficult-to-culture or harsh pathogens. We also emphasize that when WBC and PCT are used as predictive biomarkers, when the above monitoring indicators of the patients suggest a possible multiple microbial infection, 16S rDNA molecular diagnosis can be used as a supplementary testing method to achieve a more rapid identification of potential multiple microbial infections. This is the core clinical significance of this article.

Notably, both molecular approaches consistently identified *Escherichia coli* and *Staphylococcus aureus* among the predominant pathogens detected in polymicrobial infections, supporting the overall findings obtained by conventional blood culture. In addition, *Brucella* spp. were detected at a relatively higher frequency in both blood culture and 16S rDNA PCR library analysis in our cohort. This finding is consistent with the epidemiological characteristics of the Ningxia Hui Autonomous Region in Northwest China, where human brucellosis remains an important regional public health concern associated with extensive livestock farming and pastoral populations (Yin et al., 2025, Duan et al., 2026). Recent epidemiological data have also demonstrated a sustained high burden of human brucellosis in Ningxia, with *Brucella melitensis* representing the predominant circulating species. These findings suggest that the local epidemiological background may influence the spectrum of organisms encountered in molecular BSI testing. Importantly, however, the relatively frequent detection of *Brucella* spp. in the present study should be interpreted in the context of the regional disease ecology rather than considered representative of the pathogen distribution in other healthcare settings.

In the present study, the two molecular approaches were both based on 16S rDNA amplification but differed substantially in their downstream workflows. For the 16S rDNA PCR library method, cfDNA was first subjected to PCR amplification, followed by gel electrophoresis and amplicon recovery, molecular cloning, ARDRA-based colony screening, and Sanger sequencing of representative clones. In contrast, the high-throughput sequencing approach used cfDNA-derived 16S rDNA amplicons for direct sequencing on the Illumina platform. Thus, the comparison in this study represents two different strategies for analyzing 16S rDNA amplicons rather than a comparison between targeted 16S sequencing and shotgun metagenomic sequencing. Our results extend these observations by systematically comparing three methodologies, highlighting that 16S rDNA library construction not only detects more species but also preserves genetic material for downstream validation—an advantage over high-throughput sequencing, which lacks clone retention for confirmatory testing.

The observed overall percent agreement with blood culture was higher for the clone-based approach than for direct 16S amplicon sequencing. To further explore whether this difference could simply reflect a narrower analytical detection range, we compared the total number of microbial taxa detected by each method at the individual-patient level. Blood culture detected 0–1 taxa per patient, whereas the clone-based method detected 0–5 taxa, and high-throughput sequencing showed the broadest detection range, with nine patients yielding more than five taxa. These findings indicate that the higher concordance of the clone-based method with blood culture cannot be explained solely by an inability to detect additional microorganisms, because the clone-based approach identified a broader microbial spectrum than culture. One potential advantage of the clone-based workflow is that individual amplicons are physically separated through the cloning step before Sanger sequencing. This workflow may reduce redundant sequencing signals generated by highly abundant organisms and allows individual sequence reads to be traced back to representative clones. In this sense, the cloning step provides an additional layer of signal separation before sequence identification, which may contribute to a more focused microbial profile in polymicrobial specimens. By comparison, direct amplicon NGS provides substantially greater sequencing depth and can detect a broader range of microbial taxa, but this broader analytical capacity also increases the challenge of distinguishing clinically relevant microorganisms from low-abundance background signals or contaminants (Toma et al., 2014). The clinical interpretation of 16S-based NGS is also evolving with advances in library preparation and bioinformatic analysis. Current approaches increasingly incorporate optimized PCR workflows, dual-indexing strategies, contaminant-aware analytical pipelines, and improved reference databases to enhance the discrimination between clinically relevant microorganisms and background signals. However, these developments have not yet resulted in a universally standardized diagnostic threshold for clinical pathogen detection. In a multilaboratory assessment involving 90 mNGS laboratories, substantial interlaboratory variability was observed in microbial identification and reads-per-million values, particularly for low-abundance organisms, emphasizing the influence of laboratory-specific workflows and interpretation criteria on mNGS results (Han et al., 2021, Chien et al., 2022).

Bioinformatic strategies such as database preprocessing and sequence-level classification can further improve the efficiency and accuracy of microbial identification. For example, BacTag uses database preprocessing and parallel computing to facilitate gene and allele identification from sequencing data, illustrating the continuing development of computational approaches for microbial sequence interpretation. The approach demonstrated comparable or improved performance relative to several commonly used tools in artificial and clinical bacterial WGS datasets, supporting the potential value of database-aware computational strategies for bacterial identification and epidemiological analysis (Khachatryan et al., 2019). However, BacTag was primarily developed and validated for gene and allele typing from bacterial WGS data, particularly MLST-related applications, rather than for complex polymicrobial 16S rDNA amplicon datasets. Its applicability to highly heterogeneous clinical samples containing multiple bacterial taxa and low-abundance background sequences therefore requires further evaluation. In this context, the clone-based 16S rDNA PCR library approach evaluated in the present study should not be considered a substitute for modern NGS platforms. Rather, its potential value lies in the physical separation of amplified products before sequencing, relatively straightforward sequence-level traceability, and the possibility of reducing redundant mixed signals. This may provide a practical and comparatively lower-cost adjunctive strategy in selected settings where broad molecular detection is required but standardized NGS interpretation remains challenging.

In both blood culture and 16S rDNA library construction methods, a higher detection rate of *Brucella* spp. was observed, which aligns with the region’s clinical epidemiological characteristics. While high-throughput sequencing demonstrates significant concordance in pathogen detection, its inherent limitation—the absence of clinically validated diagnostic thresholds—precludes definitive differentiation between true pathogens and commensal microbiota among the numerous detected species. Consequently, authentic BSI etiologies must be interpreted by integrating clinical manifestations, host immune status, and temporal symptom profiles. This evidence positions high-throughput sequencing as adjunctive to therapeutic decision-making rather than diagnostic-grade confirmation, aligning with current consensus on molecular diagnostics in complex infections. Indeed, the practical value of integrating clinical correlation with molecular outputs is underscored by typical cases within our cohort. For instance, in an immunocompromised patient with lung adenocarcinoma presenting with negative blood cultures, the 16S library successfully rescued the detection of *Streptococcus constellatus*, which was cross-validated by a parallel wound secretion culture and indicated a clear mucosal translocation. Similarly, in a complex case of hollow viscus perforation where empirical antimicrobial therapies rendered routine cultures entirely sterile, retrospective 16S library testing successfully identified *Klebsiella pneumoniae* and *Escherichia coli*. This molecular finding not only perfectly matched the anatomical source of gastrointestinal leakage but also retrospectively validated the appropriateness of the clinician’s escalated antibiotic strategy. These real-world observations demonstrate that when routine culture fails due to antibiotic suppression, the timely supplementation of 16S molecular diagnostics provides invaluable microbiological information, provided that the data are interpreted within the context of the host’s physical barriers and clinical history.

Despite remarkable progress in diagnostic technologies, several technical limitations persist in applying advanced molecular methods for BSI diagnosis. For instance, while 16S rDNA library construction can achieve species-level identification in most clones, its reliance on universal primers introduces amplification biases, particularly against highly divergent 16S sequences, such as *Mycoplasma*. This highlights the need for more inclusive primer design to enhance the inclusivity of sequencing approaches. Moreover, the current exclusion of fungal pathogens represents a significant gap, given their critical role in polymicrobial BSI cases, especially among neutropenic patients. Integrating fungal detection methods could bridge this gap and improve diagnostic accuracy. At the same time, cost remains a substantial barrier to the widespread adoption of these advanced techniques. The per-sample sequencing expense is significantly higher than that of traditional culture-based methods, often by an order of magnitude. Another critical factor is turnaround time. In this study, after optimizing the entire process and using automated instruments, multiple samples can be tested simultaneously, and the pure experimental time can be shortened to 30–36 h. Advanced molecular diagnostics must be optimized to provide results within a timeframe that aligns with the urgency of clinical decision-making. This requires not only technical improvements but also seamless integration into existing clinical workflows. Addressing these challenges will be essential for translating next-generation sequencing technologies into routine clinical practice, ultimately improving the diagnosis and management of bloodstream infections.

The identification of *Enterobacteriaceae* and *Staphylococcaceae* as predominant pathogens in polymicrobial BSI mirrors epidemiological trends reported in large cohort studies (Bauer et al., 2020). Notably, the 50% incidence of Gram-positive/Gram-negative coinfections observed in our cohort exceeds the rates reported in European registries (Vincent et al., 2019). Although this study involves a limited sample size, the discrepancy may reflect regional variations in antimicrobial stewardship practices or healthcare-associated infection patterns. This discrepancy underscores the importance of context-specific diagnostic protocols, as our ROC analysis showed that combined testing of WBC ( > 12.54 × 10^9^/L) and PCT ( > 3.20 ng/mL) achieved an AUC of 0.927 for detecting mixed infections. These findings resonate with emerging evidence advocating biomarker panels over single parameters. For instance, Sang-Bum Hong et al. demonstrated that monocyte distribution width, combined with C-reactive protein and PCT, provides greater diagnostic value for early sepsis than either PCT or CRP alone (Woo et al., 2021). This highlights the potential of multi-parameter biomarker panels to enhance the early detection and management of critical infections. In our study, multivariate analysis further solidifies the role of WBC and PCT as independent risk factors for polymicrobial BSI. This underscores their importance in early clinical decision-making, particularly in settings where rapid and accurate diagnosis is crucial for optimizing patient outcomes. By integrating these biomarkers into diagnostic protocols, clinicians may be better equipped to identify and manage complex infections, thereby improving overall patient care.

The SIRI index (neutrophils × monocytes/lymphocytes) reflects both inflammatory intensity and immune exhaustion—a dual mechanism that may explain its predictive superiority over traditional markers. Clinically, the association between elevated SIRI/NPR and mixed infections introduces novel prognostic insights. This finding is consistent with the work of Sridhar et al., who identified the SII as an independent predictor of bloodstream infections using multivariate regression analysis (Mangalesh et al., 2023). Their study confirms that the SII can enhance the accuracy of patient assessment. However, our cohort’s median SIRI of 3.2 in polymicrobial BSI suggests that this threshold may need to be adjusted downward for infection complexity stratification. Several limitations warrant consideration. First, the single-center design and modest sample size limit generalizability, particularly with respect to pathogen distribution patterns. Second, the exclusion of viral and fungal co-infections precludes a comprehensive assessment of true polymicrobial burdens. Third, while clone retention enhances result verification, the 30–36 h turnaround time for library construction remains impractical for acute clinical decisions.

## Conclusion

In summary, this study shows that WBC and PCT are independently associated with culture-defined polymicrobial BSI and may help identify patients at increased risk of complex bloodstream infection. As an adjunct to blood culture, 16S rDNA PCR library construction extended pathogen detection and improved recognition of polymicrobial patterns while maintaining better agreement with blood culture than 16S rDNA high-throughput sequencing. These findings support the potential clinical value of adjunctive 16S-based approaches in selected patients with suspected bloodstream infection.

## Data Availability

The raw high-throughput sequencing data generated in this study have been deposited in the NCBI Sequence Read Archive (SRA) under BioProject accession number PRJNA1538502 (https://www.ncbi.nlm.nih.gov/sra/PRJNA1538502). The clone-derived 16S rRNA gene sequences have been deposited in GenBank under accession numbers QB160340–QB160436 (https://www.ncbi.nlm.nih.gov/nuccore/?term=QB160340%3AQB160436%5BACCN%5D). Additional supporting data are provided in the article and its Supplementary material.
