# Supplementary material for: Enhanced detection of polymicrobial bloodstream infections with 16S rDNA PCR library amplicons and clinical relevance
Source: Front Microbiol. 2026 Oct 15;17:1916658. doi: 10.3389/fmicb.2026.1916658 (PMC13718762; doi:10.3389/fmicb.2026.1916658)
Supplement: Supplementary file 1 [file Table_2.docx]

Supplementary Table 2 Comparison of sequencing results and blood culture results

| No. | Blood culture results | 16S rDNA test results |
| --- | --- | --- |
| 1 | streptococcus constellatus | *Klebsiella pnenmoniae*，*Escherichia coli*，streptococcus constellatus |
| 2 | coagulase-negative staphylococcus | *Klebsiella pnenmoniae*，coagulase-negative staphylococcus，*Escherichia coli*，*Serratia marcescens* |
| 3 | negatives | *Klebsiella pnenmoniae*，*Flavobacterium tumefaciens*，*Escherichia coli* |
| 4 | *Klebsiella pnenmoniae* | *Klebsiella pnenmoniae*，streptococcus granulosus，*Escherichia coli* |
| 5 | *Escherichia coli* | *Escherichia coli*，streptococcus intermedius，*Flavobacterium tumefaciens*，*Klebsiella pnenmoniae* |
| 6 | *Klebsiella penguinensis* | *Flavobacterium tumefaciens*，*Escherichia coli*，*Klebsiella penguinensis*，*Brucella*，*Acinetobacter baumannii* |
| 7 | *Escherichia coli* | *Escherichia coli*，*Flavobacterium tumefaciens*，*Brucella* |
| 8 | *Brucella* | *Aeromonas spp.*，*Flavobacterium tumefaciens*，*Brucella* |
| 9 | *Escherichia coli* | *Escherichia coli* |
| 10 | negatives | *Klebsiella pnenmoniae* |
| 11 | *Staphylococcus aureus* | *Staphylococcus aureus* |
| 12 | *Brucella* | *Brucella* |
| 13 | *Escherichia coli* | *Escherichia coli* |
| 14 | *Sputum-generating-carbon dioxide fibrophilic bacteria* | *Flavobacterium tumefaciens* |
| 15 | *Escherichia coli* | *Escherichia coli* |
| 16 | streptococcus agalactiae | streptococcus agalactiae |
| 17 | *Corynebacterium* | negatives |
| 18 | *Brucella* | *Brucella*, *Pseudomonas putida*, *Escherichia coli*, *Flavobacterium tumefaciens*，*Klebsiella pnenmoniae* |
| 19 | *Brucella* | *Brucella*，*Oslo Moraxella*，*Klebsiella pnenmoniae*，*Aeromonas spp*.，  *Aeromonas campestris* |
| 20 | *Brucella* | *Brucella*，*Flavobacterium tumefaciens* |
