# Supplementary material for: Enhanced detection of polymicrobial bloodstream infections with 16S rDNA PCR library amplicons and clinical relevance
Source: Front Microbiol. 2026 Oct 15;17:1916658. doi: 10.3389/fmicb.2026.1916658 (PMC13718762; doi:10.3389/fmicb.2026.1916658)
Supplement: Supplementary file 2 [file Table_3.docx]

Supplementary Table 3 Comparison of sequencing results

| No. | Bacteria Detected Only by 16S rDNA PCR | Bacteria Detected Only by 16S rDNA High-throughput Sequencing | Bacteria Detected by Both 16S rDNA PCR and High-throughput Sequencing |
| --- | --- | --- | --- |
| 1 | Klebsiella | - | *Escherichia coli*, *Streptococcus* |
| 2 | *Yersinia pestis*，*Klebsiella* | *Aeromonas short-wave*, *Fusobacterium*, *Streptococcus, Streptococcus*, *Staphylococcus, Brucella* | *Escherichia coli*, *Staphylococcus* |
| 3 | *Klebsiella，Corynebacterium chrysogenum* | *Streptococcus, Staphylococcus, Fusobacterium, Brucella* | *Escherichia coli* |
| 4 | *Klebsiella* | *-* | *Streptococcus, Escherichia coli* |
| 5 | *Corynebacterium chrysogenum，Klebsiella* | *-* | *Escherichia coli, Streptococcus* |
| 6 | *Klebsiella，Fusobacterium, Brucella* | *-* | *Flavobacterium aureus, Escherichia coli* |
| 7 | *-* | *Fusobacterium, Aeromonas, Staphylococcus, Streptococcus* | *Escherichia coli, Staphylococcus aureus, Brucella sp.* |
| 8 | *-* | *Fusobacterium, Staphylococcus, Streptococcus, Escherichia coli* | *Aeromonas sp. aureus, Brucella sp.* |
| 9 | *-* | *Staphylococcus, Streptococcus, Flavobacterium* | *Escherichia coli* |
| 10 | *Klebsiella* | *Fusobacterium, Aeromonas, Staphylococcus, Brucella, Flavobacterium, Escherichia coli* | *-* |
| 11 | *-* | *Fusobacteria, Escherichia coli* | *staphylococcus* |
| 12 | *-* | *Fusobacterium, Aeromonas shortwave, Staphylococcus, Staphylococcus aureus* | *Brucella* |
| 13 | *-* | *Fusobacterium, Aeromonas, Staphylococcus, Streptococcus, Flavobacterium, Brucella* | *Escherichia coli* |
| 14 | *-* | *Lactococcus, Aeromonas, Fusobacterium, Staphylococcus, Streptococcus, Escherichia coli, Brucella* | *Corynebacterium chrysogenum* |
| 15 | *-* | *Brucella, Streptococcus aureus, Streptococcus* | *Escherichia coli* |
| 16 | *-* | *Lactococcus, Flavobacterium aureus, Escherichia coli* | *streptococcus* |
| 17 | *-* | *Fusobacterium, Aeromonas, Staphylococcus, Streptococcus, Streptococcus, Flavobacterium, Brucella, Escherichia coli* | *-* |
| 18 | *Pseudomonas aeruginosa，Klebsiella* | *Lactococcus, Fusobacterium, Aeromonas, Staphylococcus, Streptococcus* | *Brucella, Flavobacterium aureus, Escherichia coli* |
| 19 | *Moraxella catarrhalis，Klebsiella* | *Lactococcus, Staphylococcus, Streptococcus, Streptococcus, Staphylococcus, Escherichia coli* | *Brucella, Fusobacterium, Aeromonas, Shortwave* |
| 20 | *-* | *Aeromonas short-wave, Streptococcus, Escherichia coli* | *Brucella, Flavobacterium* |
